# Angiotensin-I-Converting Enzyme Inhibitory Activity of Coumarins from *Angelica decursiva*

**DOI:** 10.3390/molecules24213937

**Published:** 2019-10-31

**Authors:** Md Yousof Ali, Su Hui Seong, Hyun Ah Jung, Jae Sue Choi

**Affiliations:** 1 Department of Food and Life Science, Pukyong National University, Busan 48513, Korea; yousufbge@gmail.com (M.Y.A.); seongsuhui@naver.com (S.H.S.); 2 Department of Chemistry and Biochemistry, Concordia University, Montreal, QC H4B 1R6, Canada; 3 Department of Biology, Faculty of Arts and Science, Concordia University, 7141 Sherbrooke St. W., Montreal, QC H4B 1R6, Canada; 4 Centre for Structural and Functional Genomic, Department of Biology, Faculty of Arts and Science, Concordia University, 7141 Sherbrooke St. W., Montreal, QC H4B 1R6, Canada; 5 Department of Food Science and Human Nutrition, Jeonbuk National University, Jeonju 54896, Korea

**Keywords:** *Angelica decursiva*, angiotensin-I-converting enzyme, coumarins, antihypertension, molecular docking

## Abstract

The bioactivity of ten traditional Korean *Angelica* species were screened by angiotensin-converting enzyme (ACE) assay in vitro. Among the crude extracts, the methanol extract of *Angelica decursiva* whole plants exhibited potent inhibitory effects against ACE. In addition, the ACE inhibitory activity of coumarins **1**–**5**, **8**–**18** was evaluated, along with two phenolic acids (**6**, **7**) obtained from *A*. *decursiva*. Among profound coumarins, **11**–**18** were determined to manifest marked inhibitory activity against ACE with IC_50_ values of 4.68–20.04 µM. Compounds **12**, **13**, and **15** displayed competitive inhibition against ACE. Molecular docking studies confirmed that coumarins inhibited ACE via many hydrogen bond and hydrophobic interactions with catalytic residues and zinc ion of C- and N-domain ACE that blocked the catalytic activity of ACE. The results derived from these computational and in vitro experiments give additional scientific support to the anecdotal use of *A. decursiva* in traditional medicine to treat cardiovascular diseases such as hypertension.

## 1. Introduction

Hypertension is one of the most common diseases worldwide, with many associated risk factors such as stroke, heart disease, chronic renal failure, and aneurysm [1,2]. Globally, about 25% of the adult population suffers from hypertension, and the number of people is set to rise to 29% by 2025, when a total of 1.56 billion people will be affected. Inhibition of the angiotensin-converting enzyme (ACE) is established as one modern therapeutic approach to hypertension [1]. Membrane-bound zinc (Zn) metallopeptidases ACE (EC 3.4.15.1) is a multifunctional enzyme present in the rennin–angiotensin system (RAS) that elevates blood pressure by generating the vasoconstrictor angiotensin II and degrading the vasodilator bradykinin [3]. ACE is present in many tissues, including the heart, brain, adrenal cortex, kidneys, leukocytes, alveolar macrophages, peripheral uterus, placenta, vascular tissue, liver, monocytes, and neuronal and epididymal cells, particularly in the vascular endothelial lining of the lungs [4]. Therefore, ACE inhibition is a major target in the prevention and treatment of hypertension. Many researchers have attempted to synthesize ACE inhibitors, such as captopril, enalapril, lisinopril, and fosinopril, which are all currently used as clinical antihypertensive drugs [5]. However, those drugs are often accompanied by undesirable side effects, such as persistent cough, postural hypotension, renal failure, and angioedema [6]. Extensive research has been carried out to find ACE inhibitors in natural products, which might have better drug profiles and fewer side effects. Several plant extracts and pure compounds, such as phenolics, anthraquinone, flavonoids, alkaloids, terpenoids, peptides, hydrolysable tannins, and proanthocyanidins, have been reported to inhibit ACE [7,8,9,10,11,12].

*Angelica decursiva* Fr. et Sav (Umbelliferae) is a perennial herb that grows throughout Japan, China, and Korea. It is widely employed in traditional Korean medicine to cure diseases as an antitussive, analgesic, antipyretic, and cough remedy [13,14]. In traditional Chinese medicine, it is used as a remedy for thick phlegm, asthma, and upper respiratory tract infections [15,16]. The usage of the roots of *A. decursiva* has a long history in China to clean heat, resolve summer heat, and stop bleeding [15]. During the past decade, extensive investigations have been conducted on different species of this genus. Consequently, many classes of compounds have been isolated, including different types of coumarin derivatives: Umbelliferone, 6-formyl umbelliferone, umbelliferone 6-carboxylic acid, nodakenin, nodakenetin, isorutarine, 2′-isopropyl psoralene, Pd-C-I, Pd-C-II, Pd-C-III, 4′-hydroxy Pd-C-III, columbianadin, decursin, (+)-decursidinol, decursidin, *cis*-3′-acetyl-4′-angeloylkhellactone, and 3′(*R*)-*O*-acetyl-4′(*S*)-*O*-tigloylkhellactone [13,15,17,18,19,20,21,22,23,24]. Those compounds have been reported to possess a wide range of biological activities, including antihypertensive [25], antiplatelet aggregation [26], neuroprotective [27], memory-enhancing [28], anti-amnesic [29], anti-oxidative [30], anti-inflammatory [13,24,31,32], antidiabetic, and anti-Alzheimer’s disease effects [18,19,20,21,22,23,33].

Despite the promising biological activities of *Angelica* species, no systematic studies have yet been conducted on the ACE inhibitory activities of *A. decursiva* and its coumarins. Therefore, as a part of our continuing efforts to identify potent ACE agents from natural sources, we explored the anti-hypertensive activities of *A*. *decursiva*-derived coumarin constituents. We also performed enzyme kinetic analyses of the coumarins using Lineweaver–Burk plots and secondary plots in order to confirm the type of enzymatic inhibition. The interactions between these coumarins and ACE were simulated using molecular docking analysis, and their docking energies and ACE inhibition mechanisms were examined.

## 2. Results

### 2.1. ACE Inhibitory Activity of the Selected Angelica Species

In order to evaluate the ACE inhibitory activity of *Angelica* species, the methanol (MeOH) extract of 10 different species was selected and tested using the in vitro assay. Of all these species, *A. decursiva* was found to be the most potent ACE inhibitor with an inhibition percentage (%) of 94.12 at a concentration of 163.93 µg/mL. In addition, MeOH extracts of *A. czernevia*, *A. anomala*, *A. fallax*, *A. cartilagino-marginata* var. *distans*, and *A. fallax*, showed moderate ACE activity with percentages (%) of 52.29, 50.98, 38.56, 32.35, and 31.37 at a concentration of 163.93 µg/mL (Table 1). On the other hand, *A. japonica*, *A. gigas*, *A. dahurica*, *A. anomala*, and *A. sinensis* did not show significant inhibitory activity at the concentration tested.

### 2.2. Inhibitory Activity of the Compounds Isolated from A. decursiva on ACE

**Figure 1:**
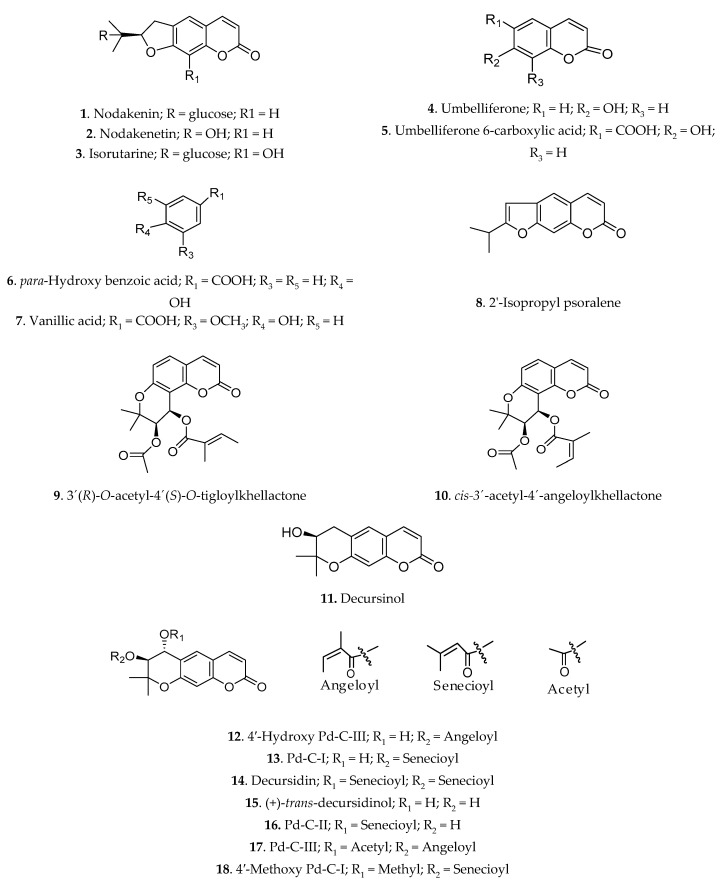
Chemical structures of the compounds isolated from *Angelica decursiva.*

To determine which of the active compounds isolated from *A. decursiva* were responsible for the inhibition of ACE, we used inhibitory assay. The ACE inhibitory activities of the compounds (nodakenin (**1**), nodakenetin (**2**), isorutarine (**3**), umbelliferone (**4**), umbelliferone 6-carboxylic acid (**5**), *para*-hydroxy benzoic acid (**6**), vanillic acid (**7**), 2′-isopropyl psoralene (**8**), 3′(*R*)-*O*-acetyl-4′(*S*)-*O*-tigloylkhellactone (**9**), *cis*-3′-acetyl-4′-angeloylkhellactone (**10**), decursinol (**11**), 4′-hydroxy Pd-C-III (**12**), Pd-C-I (**13**), decursidin (**14**), (+)-*trans*-decursidinol (**15**), Pd-C-II (**16**), Pd-C-III (**17**), and 4′-methoxy Pd-C-I (**18**)) are given in Table 2, and structures of compounds are described in Figure 1. Compounds **13** and **15** showed the highest ACE inhibitory activity among the tested compounds, with IC_50_ values of 6.75 and 4.68 µM, respectively. The positive control, captopril, had an IC_50_ value of 1.16 nM. Compounds **12**, **16**, **17**, **18**, **11**, **14**, **3**, **10**, **9**, and **2** also exhibited significant ACE inhibitory activity, with corresponding IC_50_ values of 9.41, 12.39, 15.21, 16.03, 18.29, 20.04, 68.36, 71.48, 89.36, and 102.27 µM, respectively.

### 2.3. Enzyme Kinetics in ACE Inhibition

**Figure 2:**
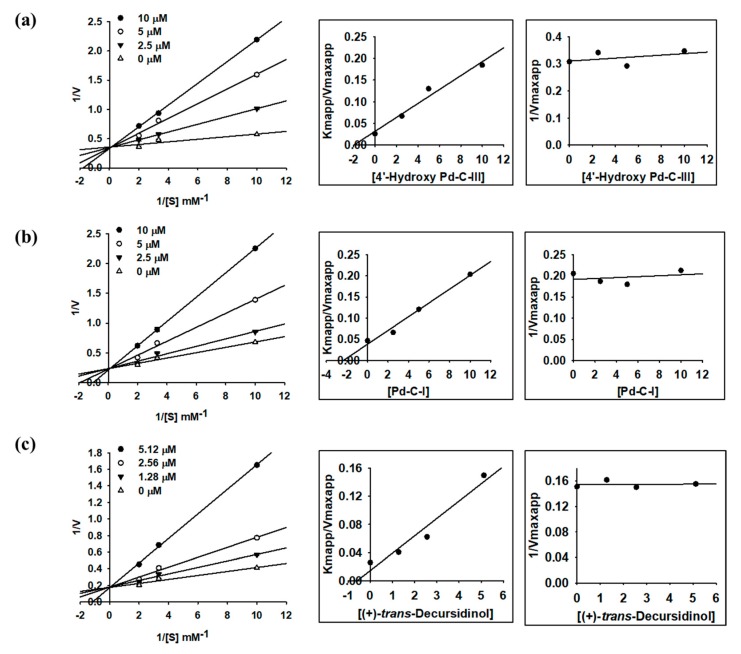
Lineweaver–Burk plots and secondary plots for ACE inhibition of *Angelica* coumarins (**a**) **12**, (**b**) **13**, and (**c**) **15**.

As part of our continuing search for coumarin derivatives from *A. decursiva*, we investigated the type of inhibition and inhibition constants (*K_i_*) of three active coumarins (**12**, **13**, and **15**) using Lineweaver–Burk and secondary plots. As shown in Figure 2, the *y*-axis intercept stayed the same, showing that the *V_max_* was a fixed value, whereas the *x*-axis intercept decreased with increasing concentrations of **12**, **13**, and **15**, suggesting that the *K_m_* increased. The secondary replot of 1/*V_max_* versus inhibitors was parallel. Thus, **12**, **13**, and **15** caused the competitive inhibition of ACE. Namely, **12**, **13**, and **15** occupied the catalytic pocket of ACE and caused a decrease in the binding affinity of ACE with substrate (FAPGG). *K_i_* values of compounds **12**, **13**, and **15** were obtained as 1.98, 2.35, and 0.59 μM, respectively (Table 3), using the secondary plot of slope (*K_m, app_*/*V_max, app_*) versus inhibitors.

### 2.4. Molecular Docking Simulation between Coumarins and ACE

**Figure 3:**
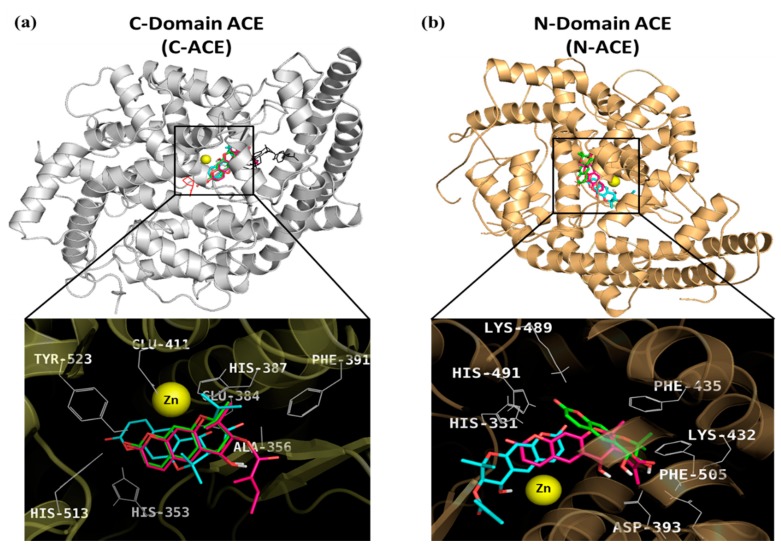
Molecular docking model for (**a**) C-ACE and (**b**) N-ACE inhibition of *Angelica* coumarins **12** (pink stick), **13** (cyan stick), and **15** (green stick), along with positive controls, captopril (red line) and FII (black line). Zinc ion (Zn) was represented as a yellow-colored sphere.

**Figure 4:**
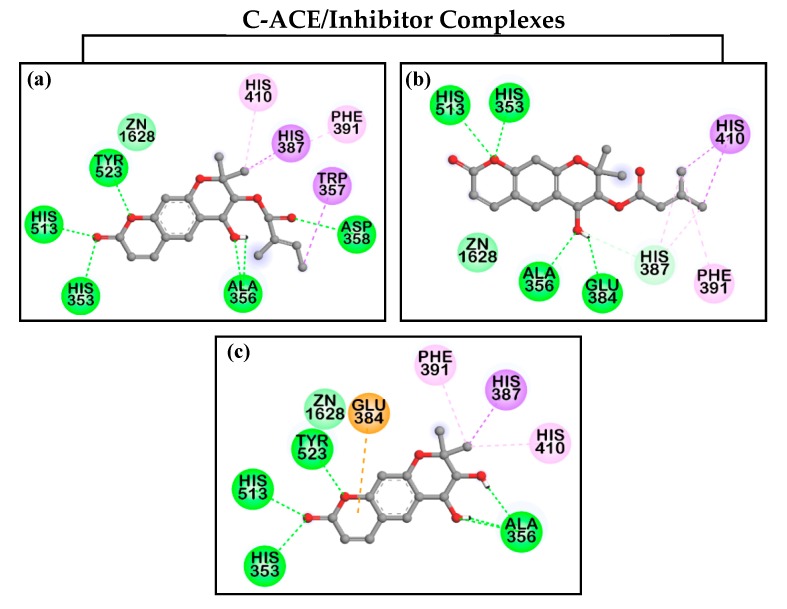

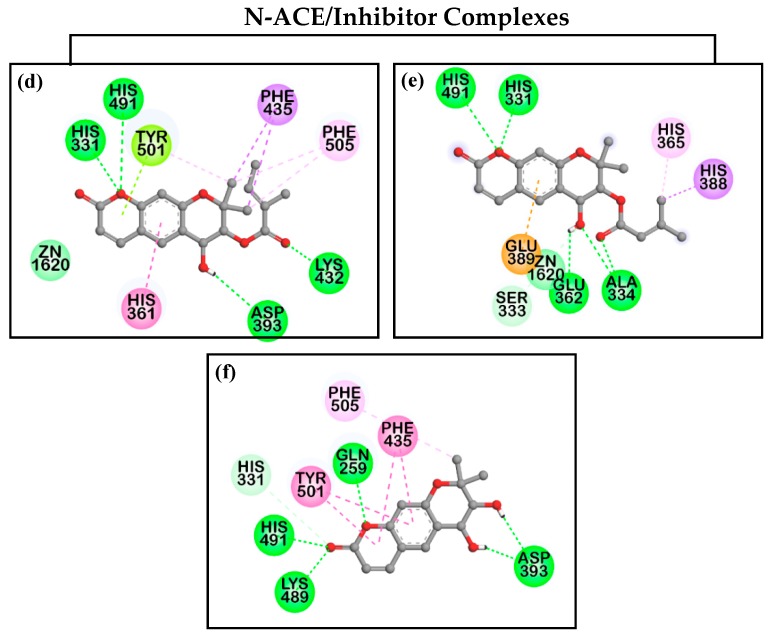
Two-dimensional (2D) diagrams of C-ACE and N-ACE inhibition of *Angelica* coumarins (**a**,**d**) **12**, (**b**,**e**) **13**, and (**c**,**f**) **15**.

Docking simulations of the interactions between the coumarins and ACE were performed at the C- and N-domains’ ACE cavity together with well-known catalytic ACE inhibitor, captopril, and reported mixed type C-ACE inhibitor, [(2*S*)-2-({3-[hydroxyl(2-phenyl-(1*R*)-1-{[(benzyloxy)carbonyl]-amino}ethyl)phosphinyl]-2-[(3-phenylisoxazol-5-yl)methyl]-1-oxo-propyl}amino)-3-(4-hydroxy-phenyl) propanoic acid] (FII) [34]. To provide a rational explanation for the significant ACE inhibition of compounds **12**, **13**, and **15**, we simulated the hydrogen bonding, hydrophobic, and electrostatic interactions between **12**, **13**, and **15** and active sites of ACE (Table 4). As shown in Figure 3, these compounds and captopril docked into the zinc-binding catalytic sites of C-ACE and N-ACE, respectively, and interacted with zinc ion via van der Waals interaction. In the docking simulation between **12** and C-ACE, six H-bonds between **12** and Ala356, Asp358, and S1′ residues, including His353, His513, and His523, were observed. In addition, four residues (His387, Trp357: Pi–Sigma interaction; Phe391, His410: Pi–Alkyl interaction) were included in hydrophobic interactions (Figure 4a). Similar to **12**, 2-ketone moiety of **15** formed two hydrogen bonds with His513 and His353 residues included in S_1_′ pocket. Asp356 residue interacted with 3′- and 4′-OH of **15** via three H-bonds. Aside from H-bonds, additional hydrophobic (Phe391, His410: Pi–Alkyl interaction; His387: Pi–Sigma interaction) and electrostatic (Glu384 residue) interactions were also detected (Figure 4c). For coumarin **13**, the binding site of **13** was closer to the zinc ion than those of **12** and **15**, whereas the number of H-bonds was less than those of **12** and **15**. (Figure 4b). The oxygen atom in position 1 of **13** formed two hydrogen bonds with His513 and His353. 4′-OH of **13** also hydrogen-bonded with Ala356 and Glu384 residues. In addition, two CH_3_ groups of senecioyl moiety in the position C3′ interacted with His410 (Pi–Sigma interaction), Phe391 (Pi–Alkyl interaction), and His387 (Pi–Alkyl interaction) residues via hydrophobic interactions.

As a result of docking analysis between tested compounds and N-ACE, oxygen atoms of **12** and **13** interacted with hydrogen atoms of His331 and His491 residues included in the S_1_′ pocket of N-ACE. These hydrogen-bonding interactions and hydrophobic interactions between 3′-substituents of inhibitors (**12** and **13**) and His and Phe residues of N-ACE make a favorable orientation to interact with zinc ion via van der Waals interaction. However, **15** interacted with His331 via carbon–hydrogen interaction, which is a relatively weak force compared to conventional hydrogen bond interaction (such as H‒O and H–N); thus **15**, could not get close enough to interact with zinc ions (Figure 3b). Our docking results indicated that **12**, **13**, and **15** could inhibit ACE by competing with substrates in the active site rather than other secondary sites (Figure 3). Moreover, **15** exhibited high selectivity for the C-ACE compared to **12** and **13**.

## 3. Discussion

Hypertension, a worldwide illness, is a major factor in cardiovascular diseases and affects a large population of adults. Some of the most effective medications for the treatment of hypertension are ACE inhibitors. Meanwhile, medicinal plants have been used for centuries to treat illnesses. Therefore, they can be important resources in developing new drug candidates. The present study demonstrated for the first time that *A*. *decursiva* and its coumarin constituents show inhibitory activity against ACE. Recently, the coumarin system found in many natural compounds has excited considerable attention. Coumarins are naturally occurring compounds widely distributed in the plant kingdom and are important components of the human diet. Coumarins have been associated with low toxicity and have garnered considerable interest due to their potentially beneficial effects on human health [35]. In recent times, coumarins have been considered a promising group of bioactive compounds that exhibit a wide range of biological activities: Anticoagulant [36], anti-inflammatory [13,22,37,38], antimicrobial [39], antioxidant [13,37], anti-allergic and antidepressant [40], antidiabetic [41], anticancer [42,43], antifungal [44], and anti-Alzheimer’s disease [18,19,20,21]. These biological activities indicate that coumarin compounds should be tested as novel therapeutic compounds. Therefore, we selected a focused collection of coumarins to increase the likelihood of finding a promising ACE inhibitor.

In a preliminary study, the MeOH extract of whole-plant *A*. *decursiva* Fr. et Sav (Umbelliferae) exhibited inhibitory effects against ACE. Recently, it was reported that another *Angelica* species, *A*. *gigas*, and its coumarin constituents showed potential antihypertensive activity through ACE inhibition [25]. Therefore, we investigated the ACE inhibition activity of *A. decursiva*-derived compounds. As illustrated in Table 2, most of the coumarins showed potent antihypertensive activity. In particular, coumarins **11**–**18** exhibited potent ACE inhibitory properties, with IC_50_ values ranging from 4.68 to 20.04 µM. Hyun et al. reported that benzopyranoids (nodakenin, umbelliferone, and umbelliferone 6-carboxylic acid) showed promising ACE inhibitory activity [25], which is similar to our results. It was also reported that the *A. decursiva* components, which are a combination of decursin, decursinol, and nodakenin, displayed potent ACE inhibitory activity [45], which is also comparable with our results. Therefore, both the present and previous investigations indicate that coumarins have potential antihypertensive activity.

Some structure–activity relationships of coumarins can be deduced. The simple coumarins in Table 2, **4** and **5** display the fundamental skeleton of coumarin derivatives and showed moderate inhibitory effects on ACE. The linear furanocoumarins, **1**–**3**, exhibited a significant inhibitory effect on ACE, whereas **8**, with two methoxy groups at the C-11 position, showed weak activity. In the series of linear and angular pyranocoumarins in Table 2, the linear pyranocoumarins were more active than the angular pyranocoumarins. Therefore, we speculate that the presence of a hydroxyl group at the 4′ or 3′ position on the chroman ring plays an important role in the ACE inhibition activity of linear pyranocoumarins **12**–**18**, and that replacement of that hydroxyl group with another functional group (angeloyl/senecioyl/acetyl/methoxy) decreases the activity. Based on our results, functional substitutions of linear pyranocoumarins on the chroman ring selectively enhance or decrease ACE inhibition activity, and a hydroxyl group seems to be important for activity. That structure–activity relationship helps us to understand the key structural elements that influence the ACE inhibitory activity of the different coumarin derivatives. A similar observation was previously reported: The presence of hydroxyl groups on the benzene ring plays a crucial role in the activity of phenolic compounds, and replacing a hydroxyl group with another functional group decreases activity [7].

In an attempt to explain the mode of ACE inhibition, we investigated enzyme kinetics analysis using two kinetic methods, the Lineweaver–Burk plot and the secondary plot, using different concentrations of FAPGG, as the substrate (0.1–0.5 mM) and inhibitor (0–10 µM). As demonstrated in Figure 2, the manner of inhibition of compounds **12**, **13**, and **15** was competitive (*K*_i_ values = 1.98, 2.35, and 0.59 µM, respectively). These results indicate that **12**, **13**, and **15** bound directly to the active site of the enzyme to prevent enzyme–substrate complex formation. Usually, lower *K*_i_ values indicate tighter binding with the enzyme and thereby greater inhibitor effectiveness. Thus, **12**, **13**, and **15** could be crucial ACE inhibitor candidates. Captopril, a competitive inhibitor, was used as the positive control [46]. Captopril was the first orally active ACE inhibitor approved to treat human hypertension [46,47].

Somatic ACE is composed of two important catalytic domains known as C- and N-domains (C-ACE and N-ACE). These two domains have been shown to exhibit different functions and different kinetic profiles. It was reported that C-ACE was responsible for most of the angiotensin-I hydrolysis, while the other key substrate, bradykinin, has seemed to be hydrolyzed by both C-ACE and N-ACE [48]. The C-ACE and N-ACE share almost 65% sequence homology with each other [49]. The catalytic sites of both ACEs were located in the middle of the enzyme and included S_1_, S_2_, S_1_′, and S_2_′ together with a Zn^2+^ metal ion [50].

To determine the molecular properties that influence the ACE inhibitory activity of the coumarins under consideration, we used AutoDock 4.2 program to run docking simulations. It was clear that the presence of certain functional groups, such as hydroxyl, ether, and ketone groups, which can act as hydrogen bond acceptors or donors, increased ACE inhibition potency. To identify a rational explanation for the significant ACE inhibition of compounds, we simulated the interactions including hydrogen bonding, hydrophobic interaction, and electrostatic interaction between compounds **12**, **13**, and **15** and key residues in both C-ACE and N-ACE. The docking of compounds **12**, **13**, and **15** at the C- and N-domains’ ACE active sites showed low docking energies (−7.86, −8.03, and −8.03 kcal/mol for C-ACE; −8.15, −8.46, and −7.98 kcal/mol for N-ACE, respectively). The remarkable ACE inhibition shown by these compounds can be explained by the two-dimensional (2D) interaction maps shown in Figure 4. Therefore, the ACE inhibitory activity of these coumarins could result from hydrogen bond interactions between hydroxyl or ketone groups and amino acids in the S_1_′ of the enzyme as well as van der Waals interaction with zinc ions that competitively block the catalytic activity of the ACE enzyme. Our docking results demonstrated that **12**, **13**, and **15** could inhibit ACE by competing with substrates in the zinc-binding active site rather than other secondary sites and were concordant with in vitro kinetic data. Moreover, **15** exhibited high selectivity for the C-ACE compared to **12** and **13**.

Previously it was reported that phenolic compounds exhibited the same mechanism for ACE inhibition as found in our study [7]. They found that the presence or absence of certain functional groups (hydroxyl, carboxyl, ketone) influenced the potential of phenolic acids to inhibit ACE activity, which is comparable to our data. It is well known that the complexes of synthetic inhibitor, captopril, interact with zinc ion and key amino acids in the active center [51]. Therefore, we suggest that the coumarins inhibit ACE activity in a manner similar to that of captopril.

A quantitative structure–activity relationship analysis showed that the number of hydroxyl groups on the benzene ring played a crucial role in activity, and that replacing hydroxyl groups with methoxy groups decreased activity [7], possibly because the hydroxyl groups in a compound form complexes with the metal ions and catalytic residues in ACE [52]. Therefore, the metal ions of ACE were reduced, and ACE activity decreased. On the other hand, the reduced ACE inhibitory activity seen with methoxy groups could have resulted from steric hindrance, which hampered the binding between the compound and the active site of ACE. Because the dihydroxanthyletin coumarins contain hydroxyl and other functional groups, their ACE inhibitory activity could result from interactions established via hydrogen bonds between hydroxyl or keto groups and amino acids in the active site that block the catalytic activity of ACE. Therefore, *A. decursiva*-derived compounds could potentially exert their antihypertensive effects primarily as ACE inhibitors. This aspect should be investigated further to clarify the beneficial and harmful effects in vivo.

## 4. Materials and Methods

### 4.1. Chemicals

ACE (1 Unit, rabbit lung), FAPGG (*N*-[3-(2-furyl)acryloyl]-Phe-Gly-Gly), and captopril were purchased from Sigma Chemical Company (St Louis, MO, USA). All chemicals and solvents used for column chromatography were of reagent grade and purchased from commercial sources, unless otherwise stated.

### 4.2. Plant Materials

MeOH extracts of whole plants of *A. decursiva* and other *Angelica* species were purchased from the Korean Plant Extract Bank, Chungcheongbuk-do, Korea, which is associated with the Korea Research Institute of Bioscience and Biotechnology. A voucher whole plant specimen (20131024) was registered and deposited in the Department of Food Science and Nutrition, Pukyong National University, Busan, Korea (Professor Choi, J.S.) for future reference.

### 4.3. Preparation of Coumarin fom A. decursiva

The following coumarins were previously isolated and identified in our laboratory: Nodakenin (**1**), nodakenetin (**2**), isorutarine (**3**), umbelliferone (**4**), umbelliferone 6-carboxylic acid (**5**), *para*-hydroxy benzoic acid (**6**), vanillic acid (**7**), 2′-isopropyl psoralene (**8**), 3′(*R*)-*O*-acetyl-4′(*S*)-*O*-tigloylkhellactone (**9**), *cis-3*′-acetyl-4′-angeloylkhellactone (**10**), decursinol (**11**), 4′-hydroxy Pd-C-III (**12**), Pd-C-I (**13**), decursidin (**14**), (+)-*trans*-decursidinol (**15**), Pd-C-II (**16**), Pd-C-III (**17**), and 4′-methoxy Pd-C-I (**18**) [13,18,19,20,21,22,53]. The structures of the isolated compounds are shown in Figure 1.

### 4.4. ACE Inhibitory Activity Assay

The ACE inhibitory activity of 18 compounds was conducted according to Hyun et al. [8] and modified to use FAPGG as the substrate. In brief, FAPGG (0.5 mM) and various concentrations of the samples were completely dissolved in 50 mM Tris–HCl buffer (pH 7.5). Twenty microliters of ACE (1 U/mL dissolved in 50 mM Tris–HCl buffer) was then mixed with 200 μL of various concentrations of the samples (1.28–163.93 μg/mL) as experimental samples or with 50 mM Tris–HCl buffer as a negative control. The inhibitory activities of the compounds were represented as percentages of inhibition in a concentration range of 1.28–163.93 μg/mL. An antihypertensive agent, captopril, was used as a positive control at a concentration of 0.06–1.63 ng/mL. All experiments were conducted three times.

### 4.5. Kinetic Parameters of the ACE Inhibition of Different Coumarins

To determine the inhibition mechanisms, ACE inhibition was evaluated by monitoring the effects of different concentrations of substrate (0.1–1 mM). The reaction mixture consisted of different concentrations of FAPGG (0.1–0.5 mM), as the substrate, and ACE in 50 mM Tris–HCl buffer. Several concentrations of each sample were added to the reaction mixture. The tested concentrations of coumarins are as follows: **12** (0, 2.5, 5, and 10 µM); **13** (0, 2.5, 5, and 10 µM); **15** (0, 1.28, 2.56, and 5.12 µM). Inhibition constants (*K_i_*) were determined by interpretation of the secondary plots from Lineweaver–Burk plot.

### 4.6. Molecular Docking Simulation in ACE Inhibition

The X-ray crystallographic structures of the C-domain and N-domain human angiotensin I-converting enzyme complex were obtained from the RCSB Protein Data Bank (PDB ID: 2XY9 and 2XYD, respectively) [34]. The protein was prepared using Accelrys Discovery Studio 16.1 (Accelrys, San Diego, CA, USA). The reported binding area between co-ligands and the protein was considered the most affirmative region for the ligand-binding docking simulation. The 2D structures of all compounds were drawn with MarvinSketch (ChemAxon, Budapest, Hungary) and converted into 3D pdb format using Chem3D Pro software (v12.0, CambridgeSoft Inc., Cambridge, MA, USA). Energy minimization of each ligand was carried out using a molecular mechanics 2 (MM2) force field. The docking analysis was conducted using AutoDock 4.2 (The Scripps Research Institute, La Jolla, CA, USA) [54]. The docking protocol for rigid and flexible ligand docking comprised 20 independent genetic algorithms. In the docking studies, selected molecules were examined to find the qualified binding poses for each compound.

### 4.7. Statistical Analysis

Statistical significance was analyzed by one-way ANOVA and Student’s *t*-test (Systat Inc., Evanston, IL, USA). All results are presented as mean ± SEM.

## 5. Conclusions

In this study, we isolated 16 coumarins and two phenolic compounds from *A*. *decursiva* and screened their ACE inhibitory activity using an in vitro ACE assay. Among these natural coumarins, **11**–**18** showed remarkable IC_50_ values of 4.68–20.04 µM. Therefore, screening and developing new ACE inhibitors from *A. decursiva* could be beneficial in the treatment of cardiovascular diseases such as hypertension. In addition, the present data indicate that linear pyranocoumarins inhibit ACE activity in vitro, and that activity against ACE and mode of action depend on the class and structure of the coumarins. These structure–function relationships could be useful for designing new ACE inhibitors based on coumarins.

## Figures and Tables

**Table 1:** Angiotensin converting enzyme (ACE) inhibitory activity of *Angelica* sp.

*Angelica* sp.	Parts	ACE Inhibitory Activity (%) ^a^
		Mean ± SEM
*Angelica japonica*	LF	−4.58 ± 0.65
*Angelica gigas*	WP	−16.34 ± 9.8
*Angelica fallax*	WP	31.37 ± 4.58
*Angelica dahurica*	ST	17.65 ± 1.31
*Angelica czernevia*	RT	52.29 ± 2.61
*Angelica dahurica*	RT	4.25 ± 2.29
*Angelica cartilagino-marginata* var. *distans*	WP	32.35 ± 0.33
*Angelica fallax*	FL	38.56 ± 3.92
*Angelica anomala*	AP	50.98 ± 1.96
*Angelica anomala*	UP	−5.23 ± 0
*Angelica sinensis*	RT	4.25 ± 0.33
*Angelica decursiva*	WP	94.12 ± 4.19
Captopril ^b^		86.27 ± 0.11

^a^ ACE inhibitory activity (%) of extracts and captopril was evaluated at the concentrations of 163.93 μg/mL and 1.63 ng/mL, respectively. ^b^ Positive control. WP, RT, LF, FL, AP, ST, and UP represent the whole plant, root, leaf, flower, aerial part, stem, and underground part, respectively.

**Table 2:** Angiotensin-converting enzyme-I (ACE) inhibitory activity of compounds isolated from *Angelica decursiva.*

Compounds	IC_50_ (µM) ^a^	Compounds	IC_50_ (µM) ^a^
**1**	112.47 ± 0.71	**11**	18.29 ± 0.61
**2**	102.27 ± 0.29	**12**	9.41 ± 0.69
**3**	68.36 ± 0.27	**13**	6.75 ± 0.43
**4**	195.55 ± 1.02	**14**	20.04 ± 0.79
**5**	156.11 ± 0.41	**15**	4.68 ± 0.22
**6**	492.44 ± 0.89	**16**	12.39 ± 0.27
**7**	839.34 ± 1.02	**17**	15.21 ± 0.39
**8**	311.09 ± 0.33	**18**	16.03 ± 0.92
**9**	89.36 ± 0.38	Captopril ^b^	0.0012 ± 0.0001
**10**	71.48 ± 0.47		

^a^ The concentration yielding 50% inhibition (IC_50_, µM) was calculated from the log dose inhibition curve and is expressed as the mean ± SEM. of triplicate experiments. ^b^ Positive control.

**Table 3:** Inhibition type and inhibition constants (*K_i_*) of compounds for ACE activity based on enzyme kinetic plots.

Test Compounds	Type of Inhibition ^a^	*K_i_* (µM) ^b^
**12**	Competitive	1.98
**13**	Competitive	2.35
**15**	Competitive	0.59

^a^ Inhibition type was determined by interpreting the Lineweaver–Burk plot and secondary plot. ^b^ The inhibition constants (*K_i_*) were determined by interpreting the secondary plot.

**Table 4:** Molecular interactions between ACE inhibitors and the ACE.

Compounds	Docked Energy (kcal/mol)	Hydrogen Bond Interaction (No. of H-bond)	van der Waals Interaction	Hydrophobic Interaction	Others
Target Enzyme: C-ACE (PDB: 2xy9)					
**12**	−7.86	His353 (1), Ala356 (2), Asp358 (1), His513 (1), Tyr523 (1)	ZN	His387, Trp357, Phe391, His410	-
**13**	−8.03	His353 (1), Ala356 (1), His513 (1), Glu384 (1), His387 (1)	ZN	His410, His387, Phe391	-
**15**	−8.03	His513 (1), Ala356 (3), Tyr523 (1), His353 (1)	ZN	Phe391, His410, His387	Glu384 (Pi–Anion)
Captopril ^a^	−8.95	Gln281 (1), His353 (1), Lys511 (1), Glu384 (1), His513 (1)	ZN	Ala354, His353, His383, Phe457, Tyr523	His383, His387 (Pi–S)
FII ^b^	−7.92	Lys118 (1), Asn66 (1), Arg522 (1), Trp59 (1)	-	Met223, Trp59, Tyr62, Trp220, Trp357, Val518, Pro519, Ala63	Arg124 (Attractive charge), Arg522 (Pi-Cation), Met223 (Pi-S)
Target Enzyme: N-ACE (PDB: 2xyd)					
**12**	−8.15	His331 (1), His491 (1), Asp393 (1), Lys432 (1)	ZN	His369, Phe505, Phe435,	Tyr501 (Pi–Lone pair)
**13**	−8.46	His331 (1), Ala334 (2), His491 (1), Glu362 (1), Ser333 (1)	ZN	His388, His365	Glu389 (Pi–Anion)
**15**	−7.98	Gln259 (1), Lys489 (1), His491 (1), Asp393 (2), His331 (1)	-	Phe435, Tyr501, Phe505	-
Captopril ^a^	−7.41	Gln259 (1), His331 (2), Lys489 (1), His491 (1), Glu362 (1), Tyr498 (1)	ZN	Ala332, His331, Tyr501	His361 (Pi–S)

^a^ Reported catalytic ACE inhibitor. ^b^ Reported peptic mixed type C-ACE inhibitor and co-ligand of 2xy9.
